# *Leptocneria vinarskii* sp. nov. (Lepidoptera: Erebidae: Lymantriinae), an overlooked Wallacean lineage of the Australian genus

**DOI:** 10.1038/s41598-017-12797-3

**Published:** 2017-09-29

**Authors:** Ivan N. Bolotov, Alexander V. Kondakov, Vitaly M. Spitsyn, Mikhail Yu. Gofarov, Yulia S. Kolosova

**Affiliations:** 1 Northern Arctic Federal University, 163002 Arkhangelsk, Russia; 2 Federal Center for Integrated Arctic Research of the Russian Academy of Sciences, 163000 Arkhangelsk, Russia

## Abstract

The tussock moth genus *Leptocneria* Butler, 1886 (Lepidoptera: Erebidae: Lymantriinae) has been considered an entirely Australian taxon that includes two species: *L. reducta* (Walker, 1855) and *L. binotata* Butler, 1886. However, we discovered a divergent lineage of *Leptocneria* inhabiting Flores Island, Lesser Sundas, Indonesia. Here, we describe this lineage as the third species of the genus, *L. vinarskii* Bolotov, Kondakov et Spitsyn sp. nov. The new species is sister to *L. reducta* but differs from it by dark gray marking patterns of the forewing that lack orange or dark yellow marks. The mean *COI* genetic distance between *L. vinarskii* sp. nov. and *L. reducta* sensu lato is 2.9%. Our findings confirm that the Wallacean region was a faunal exchange area between Sundaland and Sahul during the Pleistocene but highlight that the vicariance events may have played a crucial role in origin of the endemic faunas on the islands of East Nusa Tenggara. Additionally, we show that both Australian species most likely represent cryptic species complexes, which are in need of further taxonomic revision.

## Introduction

The genus *Leptocneria* Butler, 1886 includes two described species with an exclusively Australian distribution range^1^. The white cedar moth *Leptocneria reducta* (Walker, 1855) is famous because it is an abundant pest species, the larvae of which may cause urticarial dermatitis in humans^1–4^ and possibly abortions in mares^5^. The larvae of this species frequently defoliate white cedar trees, *Melia azedarach* ^6,7^. Additionally, these large hairy caterpillars are an important component of the diet of the Oriental Cuckoo, *Cuculus saturatus* Blyth, 1843^8^. In contrast, the biological features of the other species, *L. binotata* Butler, 1886, have not been well studied^9,10^.

Although *Leptocneria* taxa were unknown outside Australia, in the collection of the Northern Arctic Federal University (NARFU, Arkhangelsk, Russia) we discovered a sample of moth specimens from Flores Island, Lesser Sundas, Indonesia. They are related to *L. reducta*, but clearly differ from it in their marking patterns. At the first glance, we assumed that they might be recent invaders from the continent, a morphological form of *L. reducta*. However, the DNA barcoding indicates that they actually belong to a divergent mtDNA lineage that is sister to *L. reducta*. Based on these findings, we concluded that this lineage is a distinct Wallacean species, and it is described herein. We also show that the Australian species most likely represent two complexes of cryptic species-level taxa, but their in-depth revision is beyond the scope of the present study.

## Results

**Figure 1:**
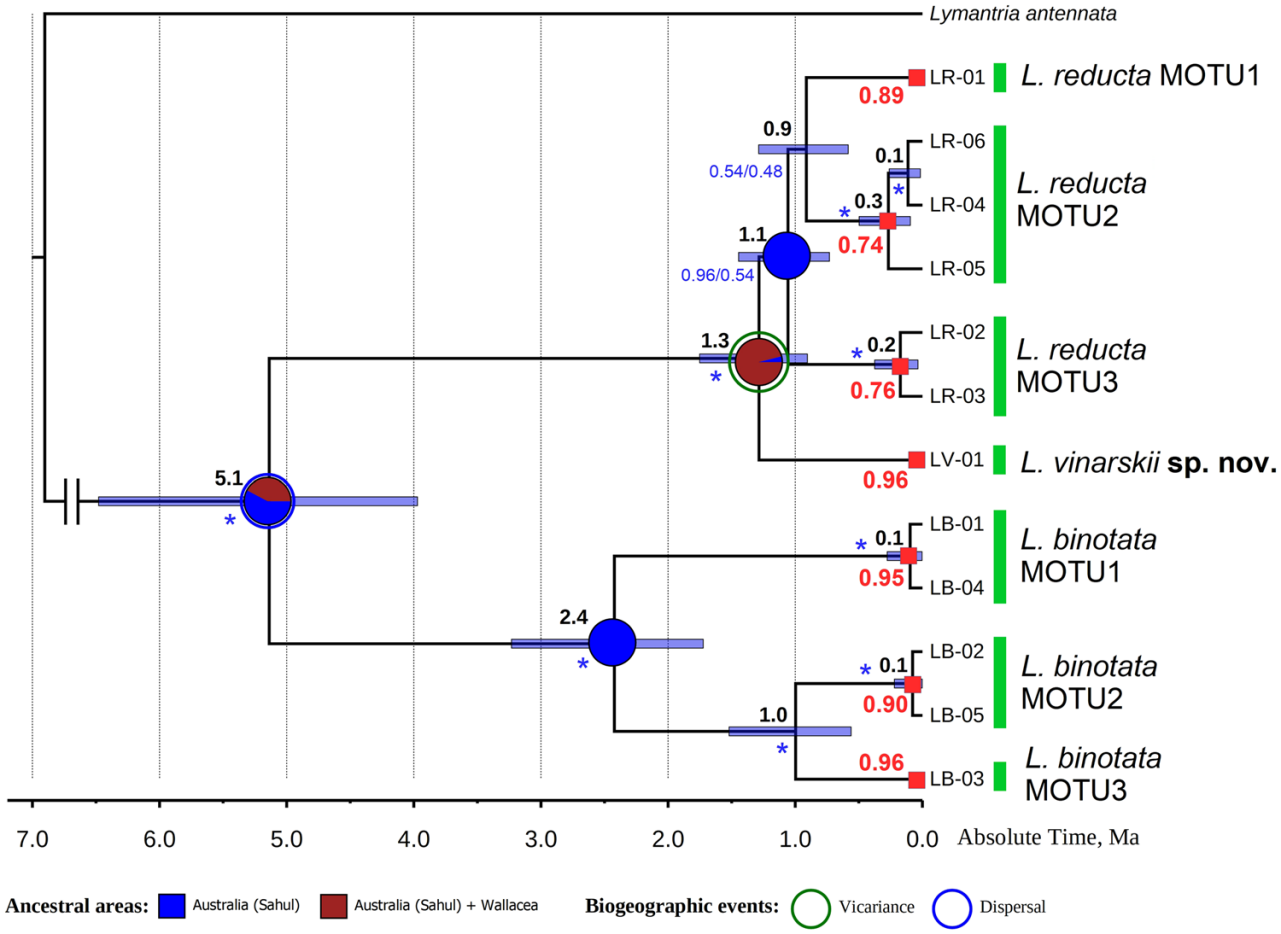
Biogeography and divergence times of the genus *Leptocneria* Butler, 1886 inferred from statistical analyses. The ultrametric chronogram was calculated under a lognormal relaxed clock model and a Yule speciation model implemented in BEAST 2.4.6 and was obtained for the *COI* dataset with 12 in-group haplotypes (see Supplementary Table 1 for details). Pie chaps near nodes indicate the probabilities of certain ancestral areas with respect to combined results under two different modeling approaches (S-DIVA and S-DEC). Black numbers near nodes are the mean age values, and bars are 95% confidence intervals of the estimated divergence time between lineages (Ma). A haplotype of *Lymantria antennata* was used as an out-group. Blue numbers near branches are Bayesian posterior probabilities inferred from MrBayes/BEAST (an asterisk indicates BPP ≥ 0.95). Solid red numbers near nodes are probabilities of species-level MOTUs (red squares) based on the highest Bayesian supported solution of the PTP species delimitation model.

**Figure 2:**
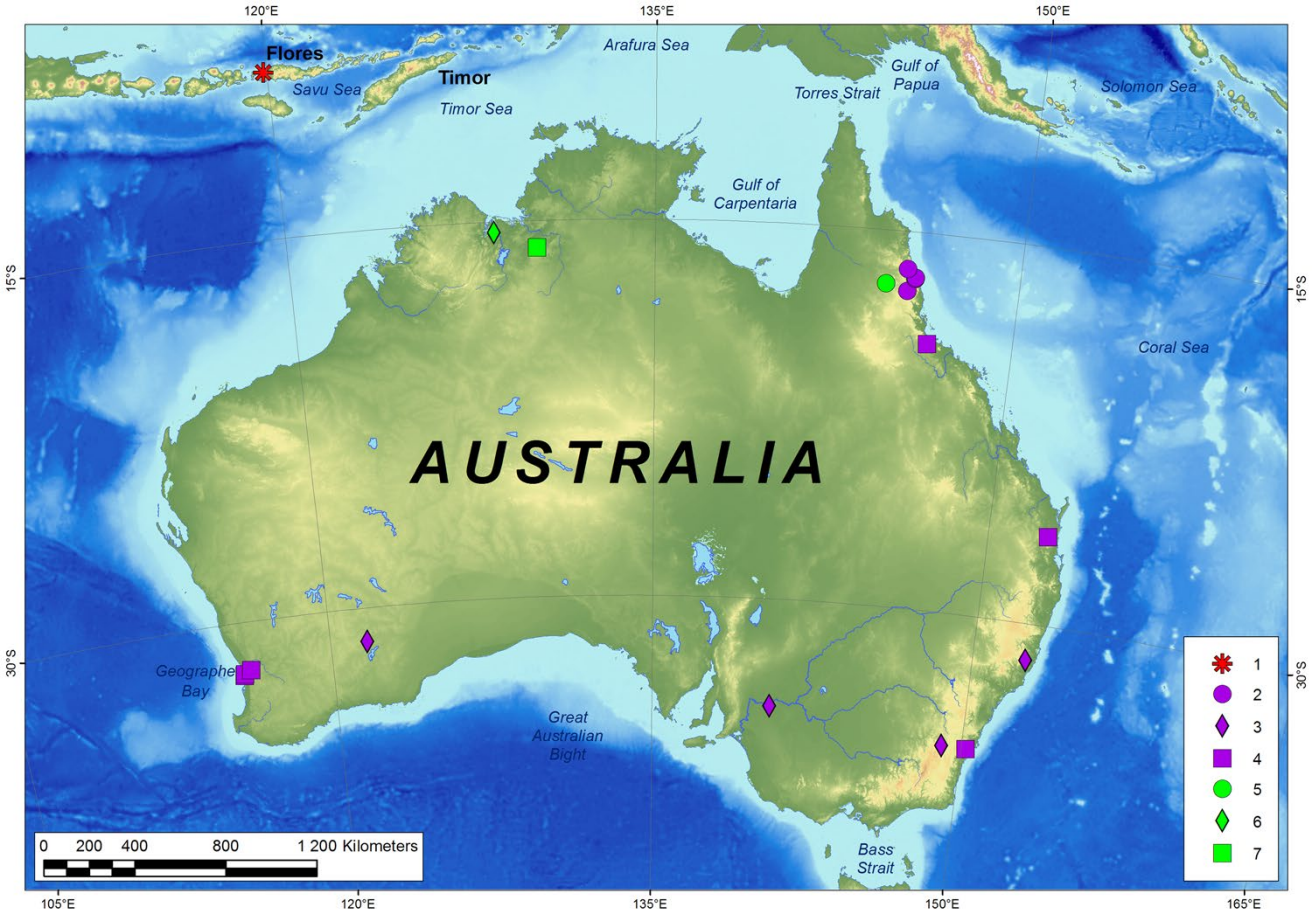
Records of mtDNA lineages of *Leptocneria* spp. based on the DNA barcoding data (see Supplementary Table 1): *L. vinarskii* Bolotov, Kondakov et Spitsyn **sp. nov**. (1), *L. reducta* MOTU1 (2), *L. reducta* MOTU3 (3), *L. reducta* MOTU2 (4), *L. binotata* MOTU1 (5), *L. binotata* MOTU3 (6), and *L. binotata* MOTU2 (7). The map was created using ESRI ArcGIS 10 software (www.esri.com/arcgis); the topographic base of the map was created with Natural Earth Free Vector and Raster Map Data (www.naturalearthdata.com) and General Bathymetric Chart of the Oceans (www.gebco.net). Map: Mikhail Yu. Gofarov.

The three sequenced specimens from Flores Island share a single haplotype of the *cytochrome c oxidase subunit I* (*COI*) gene (Supplementary Table 1). The mean *p*-distances between this haplotype and other taxa in the genus *Leptocneria* are illustrated in Table 1. The Bayesian phylogenetic analysis reveals that the haplotype from Flores is sister to a large clade that contains the *L. reducta* sensu lato haplotypes (Fig. 1 and Supplementary Fig. 1). The Bayesian species delimitation analysis suggests that the two species from Australia, i.e., *L. reducta* and *L. binotata*, both represent species complexes, each of which comprises three molecular operational taxonomic units (MOTUs), and the haplotype from Flores belongs to a separate MOTU (Fig. 1). The mean *p*-distances between the MOTUs within each clade vary from 2.7 to 5.9% (Table 1). Most MOTUs share distinct distribution records that correspond to the allopatric speciation model (Fig. 2).

**Table 1:** Genetic divergences (mean uncorrected *p*-distance ± standard error, %) between taxa in the genus *Leptocneria* Butler, 1886.

Taxon	*L. reducta* MOTU1	*L. reducta* MOTU2	*L. reducta* MOTU3	*L. binotata* MOTU1	*L. binotata* MOTU2	*L. binotata* MOTU3
*L. reducta* MOTU2	2.7 ± 0.7					
*L. reducta* MOTU3	3.4 ± 0.7	2.7 ± 0.7				
*L. binotata* MOTU1	10.2 ± 1.3	10.2 ± 1.3	10.5 ± 1.3			
*L. binotata* MOTU2	10.4 ± 1.3	10.8 ± 1.4	10.4 ± 1.3	5.9 ± 1.0		
*L. binotata* MOTU3	10.3 ± 1.3	10.4 ± 1.3	10.1 ± 1.3	5.4 ± 1.0	2.9 ± 0.7	
***L. vinarskii*** **sp. nov**.	**3.0 ± 0.7**	**2.9 ± 0.7**	**2.8 ± 0.7**	**9.6 ± 1.3**	**9.6 ± 1.3**	**9.7 ± 1.3**

The standard error of each mean distance was assessed using a bootstrap approach with 1000 replicates. The distance values between *L. vinarskii* **sp. nov**. and the other *Leptocneria* taxa are in bold.

With respect to our combined biogeographic model and time-calibrated *COI* phylogeny (Fig. 1), the MRCA of *L. vinarskii* and Australian taxa of *L. reducta* complex appears to be continuously ranged in East Nusa Tenggara and Australia in the mid-Pleistocene with subsequent separation via a vicariance event (probability 96.1%; mean age 1.3 Ma, 95% HPD 0.9–1.7 Ma). Each separate biogeographic model (S-DIVA and S-DEC) also support the vicariance scenario (Supplementary Fig. 2). The origin of the crown group of the genus was placed near the Miocene – Pliocene boundary (mean age 5.1 Ma, 95% HPD 4.0–6.5 Ma). The combined scenario suggests that the *Leptocneria* MRCA originated somewhere in Australia or in Australia + Wallacea (probability 57.9% for Australia and 42.1% for Australia + Wallacea). The S-DEC model supports the same scenario (probability 56.8% for Australia and 43.2% for Australia + Wallacea), whereas the S-DIVA model indicates the possible primary role of Australia (probability 72.5%) (Supplementary Fig. 2).

Taxonomy

Family Erebidae Leach, [1815]

Subfamily Lymantriinae Hampson, 1893

Genus *Leptocneria* Butler, 1886

Type Species: *Leptocneria binotata* Butler, 1886

*Leptocneria vinarskii* Bolotov, Kondakov et Spitsyn **sp. nov**.

**Figure 3:**
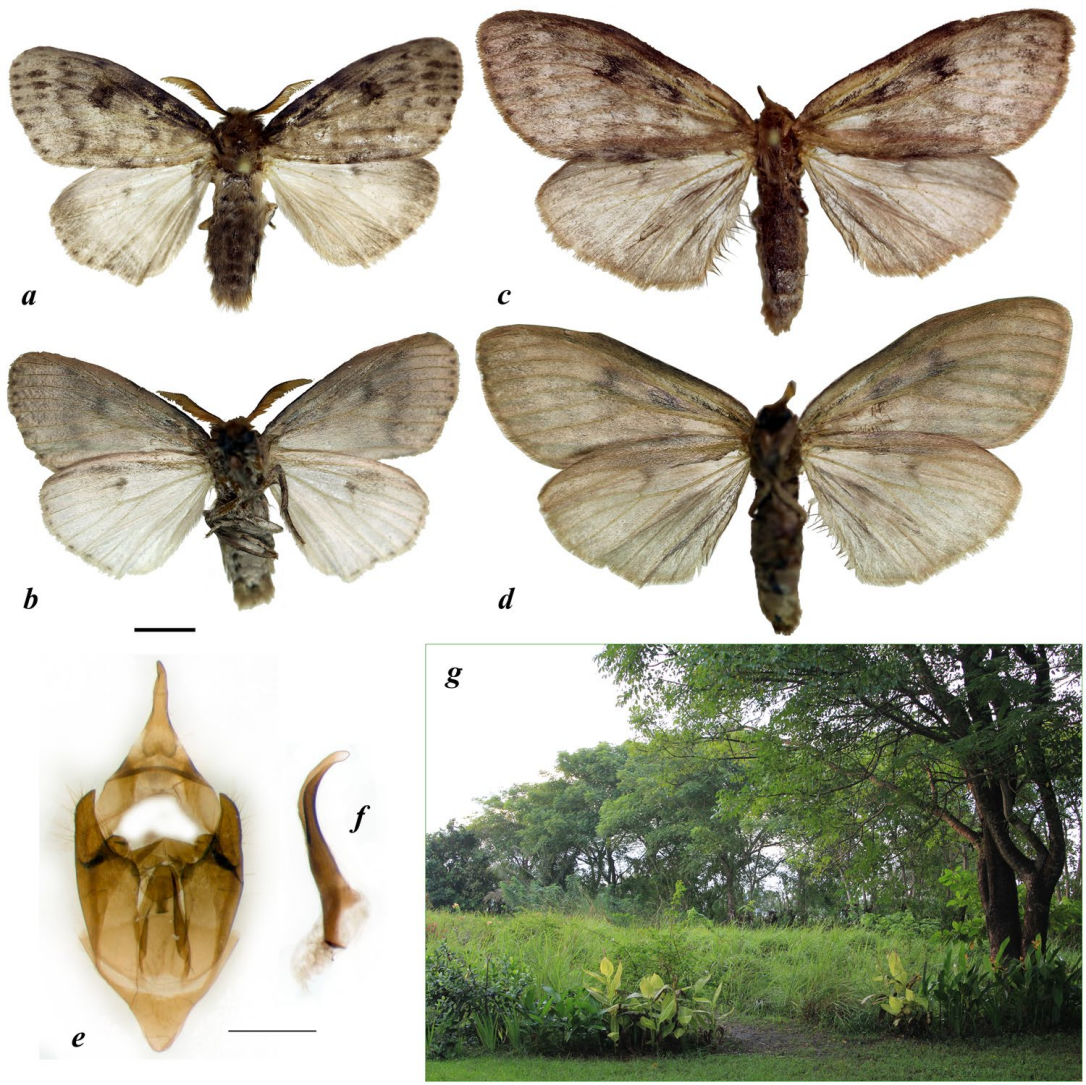
*Leptocneria vinarskii* Bolotov, Kondakov et Spitsyn sp. nov. Holotype male (specimen no. Sph0589, reference *COI* sequence no. MF036688): (**a**) upper side, and (**b**) underside. Paratype female (specimen no. Sph588, reference *COI* sequence no. MF036689): (**c**) upper side, and (**d**) underside (scale bar = 5 mm). Male genitalia (holotype): (**e**) genitalia, and (**f**) aedeagus (scale bar = 1 mm). (**g**) Type locality: Labuan Bajo, Komodo Ecolodge, garden and grasslands on the sea coast. Photos: Vitaly M. Spitsyn (**a–f**) and Yulia S. Kolosova (**g**).

Type material. Holotype male, INDONESIA, Lesser Sundas, East Nusa Tenggara, Flores Island: Labuan Bajo, Komodo Ecolodge, 8°31’21”S, 119°52’16”E, garden and grasslands on the sea coast, 13–20.i.2015, local coll. leg. (NARFU, voucher no. Sph0589). Paratypes: 3♀, INDONESIA, Lesser Sundas, East Nusa Tenggara, Flores Island: Labuan Bajo, Komodo Ecolodge, garden and grasslands on the sea coast, 8°31’21”S, 119°52’16”E, 13–24.i.2015, local coll. leg. (NARFU, voucher nos. Sph588, Sph700, and Sph701) (Fig. 3).

DNA barcoding: Reference sequences in GenBank: MF036687, MF036688, and MF036689. The mean *COI p*-distance between the new species and *L. reducta* sensu lato is 2.9 ± 0.6%, and that between the new species and *L. binotata* sensu lato is 9.6 ± 1.3% (Table 1).

Etymology. This new species is dedicated to Dr. Maxim V. Vinarski, a well-known Russian zoologist.

Diagnosis. The new species is similar to *L. reducta* sensu lato but differs from it by dark gray marking patterns of the forewing, which lack orange or dark yellow marks.

Description. *Head*: Male and female antennae bipectinate. Eyes black, without hairs. Frons gray. Labial palpi longer than eye diameter, dark gray. *Thorax*: Thorax and legs uniformly dark gray. Forewing length: male 18 mm; female 23–26 mm. Upper side of male forewing gray, with dark gray markings: unclear marks between veins in marginal area, diffused zigzag postdiscal line, large rounded discal spot, broadly dark along costal area. Upper side of male hindwing light gray, with unclear rounded gray discal spot, darkness in apical area and unclear grey marks between veins in marginal area. Underside of male forewing gray, with dark gray marks between veins in marginal area, broadly dark in postdiscal and costal areas, with large diffuse discal spot. Underside of male hindwing light gray, with vague rounded gray discal spot, and small gray spots between veins in marginal area. Upper side of female forewing gray, with diffuse dark gray markings: vague marks between veins in marginal area, large discal and postbasal spots, and narrowly dark along costal and dorsal margins. Upper side of female hindwing gray, with small, diffuse discal spot. Undersides of female wings uniformly gray, slightly dark in cell of the forewing. Fringes of both wings gray. *Abdomen*: Uniformly dark gray. *Male genitalia:* Uncus long, straight, tapering posteriorly to pointed tip. Valva short, with broad basal half and narrow, acuminate distal half. Saccus conical. Aedeagus long, strongly concave, expanded at base. Coremata absent. *Female genitalia:* not examined.

Distribution. West Flores; known only from the type locality, but may also inhabit other East Nusa Tenggara islands (e.g., Timor, Sumba, and Sumbawa), whose lepidopteran faunas are poorly known.

Habitat. Lowland semi-natural habitats near the sea coast.

## Discussion

Our results reveal that the number of species in the genus *Leptocneria* was largely underestimated, because each of the previously described species in the genus most likely represents a species complex (Fig. 1 and Supplementary Fig. 1). Although a revision of the cryptic taxa from Australia is well beyond the scope of this study, we could suggest that at least part of the lineages most likely arose via allopatric speciation driven by some inland barriers, e.g., continuous desert areas (Fig. 2). However, we discovered that the distributional range of this genus, which was considered endemic to Australia^1^, extends into the islands of East Nusa Tenggara. The distant lineage from Flores Island belongs to *L. vinarskii* **sp. nov**., a species that is new to science (Fig. 3). This lineage shares a common ancestor with *L. reducta* sensu lato, which most likely spread to East Nusa Tenggara from Australia through the drying Sahul Shelf (Fig. 1). In accordance with the results of our biogeographic modeling and time-calibrated phylogenetic analyses, we could suggest that these taxa were likely separated via a vicariance event in the mid-Pleistocene, approximately 1.3 Ma ago. The ancestral area of the *Leptocneria* MRCA remains uncertain, because different models placed it in Australia or in Australia + Wallacea, although the primary role of the Sahul region for subsequent diversification of the genus is not in doubt.

The lepidopteran fauna of the Lesser Sunda Islands is poorly known. A few available sources reveal that the faunas of these islands are largely of Asian origin^11–14^. Examples of moth taxa with clear Asian affinities inhabiting the East Nusa Tenggara Islands are common among the Erebidae, Lycaenidae, Papilionidae, and other families^11,15–19^. In contrast, the spread of Australian moth taxa into Wallacea is less well-known, although examples have been recorded among the Sphingidae^20^ and Lasiocampidae^21,22^. The biogeographic analysis of birdwing butterflies (Papilionidae) suggests that Wallacea was the source of numerous dispersal events towards neighboring areas (Sahul and Sunda)^11^. Our novel discovery confirms that the Wallacean region was a faunal exchange area between Sundaland and Sahul during the Pleistocene^14^, but highlights that the vicariance events may have played a crucial role in origin of the endemic faunas on the islands of East Nusa Tenggara.

## Methods

### Taxon sampling and laboratory protocols

The sequence data set that combine our materials and published data includes a total of 48 sequences of *Leptocneria* spp. (Supplementary Table 1). Available *COI* sequences were obtained from the Barcoding of Life Identification System (BOLD IDS) database and from NCBI’s GenBank^23,24^. The majority of these specimens was sequenced under a comprehensive analysis of Lepidoptera from the Australian National Insect Collection^25^. For molecular analyses, we used three specimens of *L. vinarskii* Bolotov, Kondakov et Spitsyn **sp. nov**. from the collection of the Northern Arctic Federal University (NARFU), Arkhangelsk, Russia. The total DNA was extracted from a single leg of each dry specimen using a standard phenol/chloroform procedure^26^. The standard primers LepF and LepR were used for the amplification of 660-bp-long barcode fragments of the *COI* gene^27^. The PCR mix contained approximately 200 ng of total cellular DNA, 10 pmol of each primer, 200 μmol of each dNTP, 2.5 μl of PCR buffer (with 10 × 2 mmol MgCl_2_), 0.8 units Taq DNA polymerase (SibEnzyme Ltd., Novosibirsk, Russia), and H_2_O added for a final volume of 25 μl. Thermocycling included one cycle at 95 °C (4 min), followed by 38–40 cycles of 95 °C (50 sec), 50 °C (50 sec), and 72 °C (50 sec) and a final extension at 72 °C (5 min). Forward and reverse sequencing was performed on an automatic sequencer (ABI PRISM® 3730, Applied Biosystems) using an ABI PRISM® BigDye™ Terminator v. 3.1 reagent kit. Resulting sequences were checked manually using a sequence alignment editor (BioEdit version 7.2.5)^28^.

### Sequence alignment and phylogenetic analysis

The alignment of sequences was performed using the ClustalW algorithm of MEGA6^29^. For phylogenetic analyses, the sequence data set was collapsed into 12 unique *COI* haplotypes of *Leptocneria* spp. (657 bp in length) using an online FASTA sequence toolbox, FaBox v. 1.41^30^, with subsequent checking via a *p*-distance matrix of MEGA6 (we used uncorrected pairwise genetic distances)^29^. As an out-group, a haplotype of *Lymantria antennata* Walker, 1855 was used (Supplementary Table 1). The lacking sites were treated as missing data. The best models of sequence evolution as suggested by the corrected Akaike Information Criterion of MEGA6^29^ were as follows: 1^st^ codon of *COI*: TN93+G (G = 0.31), 2^nd^ codon of *COI*: TN93, and 3^rd^ codon of *COI*: HKY+I (I = 0.12). Phylogenetic relationships were reconstructed based on Bayesian inference implemented in MrBayes v. 3.2.6^31^. The analyses were performed using the following parameters: nchains = 4, nruns = 2, samplefreq = 1000, temp = 0.1; 10% of the sampled trees were discarded as burn-in (pre-convergence part). Runs were conducted for 3 million generations. Convergence of the MCMC chains to the stationary distribution was checked visually based on the plotted posterior estimates using a MCMC trace analysis tool (Tracer v1.6)^32^. Calculations were performed at the San Diego Supercomputer Center through the CIPRES Science Gateway^33^.

### Species delimitation

To delimit prospective species-level units, we used a molecular approach based on the concept of MOTUs^34,35^. The MOTUs were separated using the Poisson Tree Processes (PTP) model to infer putative species boundaries on a phylogenetic input tree inferred from a Bayesian analysis of the *COI* haplotype sequences^36^. We used a Bayesian implementation of the PTP model for species delimitation through an online bPTP server (http://species.h-its.org/ptp) with 100,000 MCMC generations and 10% burn-in^36^. The out-group haplotype was removed from the input tree using an appropriate option of the server.

### Divergence time estimates

We estimated the acceptance of a molecular clock approach to our multi-gene data set using the Tajima’s relative rate test of MEGA6^29^, which is not reject the null hypothesis of equal rates between lineages (*P* > 0.05 in all possible combinations). Hypothetical divergence times were estimated in BEAST 2 v. 2.4.6 using a lognormal relaxed clock algorithm with the Yule speciation model as the tree prior^37^. Calculations were performed at the San Diego Supercomputer Center through the CIPRES Science Gateway^33^. We specified similar settings to three partitions (3 codons of *COI*) as in the MrBayes analyses (see above). To dating the phylogeny, a substitution rate of 1.78% per million years for *COI* was applied, which is the most reliable estimation of the mean evolutionary rate in insects^38^. Four replicate searches were conducted, each with 25 million generations. The trees were sampled every 1,000th generation. The log files were checked visually with Tracer v. 1.6 for an assessment of the convergence of the MCMC chains and the effective sample size (ESS) of parameters^32^. All ESS values were recorded as >400; the posterior distributions were similar to the prior distributions. The resulting tree files from four independent analyses were compiled with LogCombiner v. 2.1.3^37^. The first 10% of trees were discarded as an appropriate burn-in. The maximum clade credibility tree was obtained by using TreeAnnotator v. 2.1.3^37^.

### Ancestral area reconstructions

We tested ancestral area patterns using two different approaches, *i.e*., Statistical Dispersal-Vicariance Analysis (S-DIVA) and Statistical Dispersal-Extinction Cladogenesis (S-DEC) implemented in RASP v. 3.2^39^. For the ancestral area reconstruction, we used the set of 90,004 post-burn-in binary trees that were combined from four runs of BEAST v. 2.4.6 (see above). As a condensed tree, we used the user-specified consensus tree, which was obtained based on this set of trees using TreeAnnotator v. 2.1.3 (see above). From both of the tree data sets, out-group sequence was removed using the appropriate option of RASP v. 3.2. We coded two possible distribution areas of the in-group taxa as follows: (a) Australia (Sahul), and (b) Wallacea. The S-DIVA models were calculated with the following parameters: max areas = 2; allow reconstruction with max reconstructions = 100; max reconstructions for final tree = 1000; and allowing extinctions. The S-DEC analyses were run with default settings and max areas = 2. In addition to the evaluations obtained from each analysis separately, we used generalized results of the two modeling approaches, which were combined using an algorithm implemented in RASP v. 3.2.

### Nomenclatural acts

The electronic edition of this article conforms to the requirements of the amended International Code of Zoological Nomenclature, and hence the new name contained herein is available under that Code from the electronic edition of this article. This published work and the nomenclatural acts it contains have been registered in ZooBank (http://zoobank.org/), the online registration system for the ICZN. The LSID for this publication is: urn:lsid:zoobank.org:pub:435C007C-6B18-4953-9BE5-5823F502D613. The electronic edition of this paper was published in a journal with an ISSN, and has been archived and is available from PubMed Central.

### Data availability

The *COI* sequences generated during this study are available from GenBank. Accession number for each specimen is presented in Supplementary Table 1. The type specimens of the new species are available in the collection of the Northern Arctic Federal University (NARFU), Arkhangelsk, Russia (voucher nos. Sph0589 [holotype], Sph588, Sph700, and Sph701 [paratypes]).

## Electronic supplementary material

Supplementary Information
