# Supplementary material for: Leptocneria vinarskii sp. nov. (Lepidoptera: Erebidae: Lymantriinae), an overlooked Wallacean lineage of the Australian genus
Source: Sci Rep. 2017 Sep 29;7:12430. doi: 10.1038/s41598-017-12797-3 (PMC5622091; doi:10.1038/s41598-017-12797-3)
Supplement: Supplementary file 1 — Supplementary Information [file 41598_2017_12797_MOESM1_ESM.pdf]

Leptocneria vinarskii **sp. nov.** (Lepidoptera: Erebidae: Lymantriinae), an overlooked Wallacean lineage of the Australian genus

Ivan N. Bolotov, Alexander V. Kondakov, Vitaly M. Spitsyn, Mikhail Yu. Gofarov & Yulia S. Kolosova

Supplementary Table 1. List of the mitochondrial *COI* sequences examined in the present study

Species	Haplotype Code	GenBank/ BOLD IDS acc. no.	Sample Code	Locality	Latitude	Longitude	Source**
Leptocneria vinarskii **sp. nov.**	LV-01	MF036688	Holotype: Sph0589 (male)	Indonesia: Flores Island, Labuan Bajo	-8.522	119.871	This study
L. vinarskii **sp. nov.**	LV-01	MF036687	Paratype: Sph0700 (female)	Indonesia: Flores Island, Labuan Bajo	-8.522	119.871	This study
L. vinarskii **sp. nov.**	LV-01	MF036689	Paratype: Sph0588 (female)	Indonesia: Flores Island, Labuan Bajo	-8.522	119.871	This study
L. reducta MOTU1	LR-01	ANIC336-06	ANIC Gen No. 000528	Australia: Queensland	-16.480	145.380	BOLD IDS
L. reducta MOTU1	LR-01	ANICE513-10	10ANIC-01510	Australia: Queensland	-17.350	145.430	BOLD IDS
L. reducta MOTU1	LR-01	LOQ226-04	04HBL004226	Australia: Queensland	-16.850	145.717	BOLD IDS
L. reducta MOTU1	LR-01	LOQ228-04	04HBL004228	Australia: Queensland	-16.850	145.717	BOLD IDS
L. reducta MOTU1	LR-01	LOQ337-04	04HBL004337	Australia: Queensland	-16.850	145.717	BOLD IDS
L. reducta MOTU1	LR-01	LOQC715-05	05-QLD-00715	Australia: Queensland	-16.810	145.730	BOLD IDS
L. reducta MOTU3	LR-02	LNSWE543-06	06-NSWE-00543	Australia: New South Wales	-31.459	152.476	BOLD IDS
L. reducta MOTU3	LR-02	LNSWF102-06	06-NSWE-01042	Australia: New South Wales	-31.459	152.476	BOLD IDS
L. reducta MOTU3	LR-02	LNSWF586-06	06-NSWE-01526	Australia: New South Wales	-31.459	152.476	BOLD IDS
L. reducta MOTU3	LR-02	LNSWF616-06	06-NSWE-01556	Australia: New South Wales	-31.459	152.476	BOLD IDS
L. reducta MOTU3	LR-02	LNSWF630-06	06-NSWE-01570	Australia: New South Wales	-31.459	152.476	BOLD IDS
L. reducta MOTU3	LR-02	LNSWF694-06	06-NSWE-01634	Australia: New South Wales	-31.459	152.476	BOLD IDS
L. reducta MOTU3	LR-02	LNSWG078-06	06-NSWE-01958	Australia: New South Wales	-31.459	152.476	BOLD IDS
L. reducta MOTU3	LR-02	LNSWG303-06	06-NSWE-02183	Australia: New South Wales	-31.459	152.476	BOLD IDS
L. reducta MOTU3	LR-02	LNSWG315-06	06-NSWE-02195	Australia: New South Wales	-31.459	152.476	BOLD IDS
L. reducta MOTU3	LR-02	LNSWG316-06	06-NSWE-02196	Australia: New South Wales	-31.459	152.476	BOLD IDS
L. reducta MOTU3	LR-02	LNSWG331-06	06-NSWE-02211	Australia: New South Wales	-31.459	152.476	BOLD IDS
L. reducta MOTU3	LR-03	PHLCD1241-12	BIOUG02106-G08	Australia: Australian Capital Territory	-35.261	149.059	BOLD IDS
L. reducta MOTU3	LR-03	PHLCD1649-12	BIOUG02111-B01	Australia: Australian Capital Territory	-35.261	149.059	BOLD IDS
L. reducta MOTU3	LR-03	PHLCD1650-12	BIOUG02111-B02	Australia: Australian Capital Territory	-35.261	149.059	BOLD IDS
L. reducta MOTU3	LR-03	PHLCD1662-12	BIOUG02111-C02	Australia: Australian Capital Territory	-35.261	149.059	BOLD IDS
L. reducta MOTU3	LR-03	PHLCD1671-12	BIOUG02111-C11	Australia: Australian Capital Territory	-35.261	149.059	BOLD IDS

Species	Haplotype Code	GenBank/ BOLD IDS acc. no.	Sample Code	Locality	Latitude	Longitude	Source**
L. reducta MOTU3	LR-03	PHSAU740-12	BIOUG02217-C08	Australia: South Australia	-34.274	140.640	BOLD IDS
L. reducta MOTU3	LR-03	WALPA2367-12	BIOUG04371-H03	Australia: Western Australia	-31.347	121.715	BOLD IDS
L. reducta MOTU2	LR-04	WALPA4911-13	BIOUG05055-F06	Australia: Western Australia	-31.936	116.180	BOLD IDS
L. reducta MOTU2	LR-04	WALPA4912-13	BIOUG05055-F07	Australia: Western Australia	-31.936	116.180	BOLD IDS
L. reducta MOTU2	LR-04	WALPA4913-13	BIOUG05055-F08	Australia: Western Australia	-31.936	116.180	BOLD IDS
L. reducta MOTU2	LR-04	WALPA6088-13	BIOUG06112-B05	Australia: Western Australia	-32.066	115.852	BOLD IDS
L. reducta MOTU2	LR-04	WALPA6108-13	BIOUG06112-D01	Australia: Western Australia	-32.133	115.832	BOLD IDS
L. reducta MOTU2	LR-04	WALPA6109-13	BIOUG06112-D02	Australia: Western Australia	-32.133	115.832	BOLD IDS
L. reducta MOTU2	LR-04	WALPA6110-13	BIOUG06112-D03	Australia: Western Australia	-32.066	115.852	BOLD IDS
L. reducta MOTU2	LR-04	WALPA6111-13	BIOUG06112-D04	Australia: Western Australia	-32.066	115.852	BOLD IDS
L. reducta MOTU2	LR-05	LOQT728-06	gvc6432-1L	Australia: Queensland	-19.381	146.449	BOLD IDS
L. reducta MOTU2	LR-05	LOQT730-06	gvc3363-1L	Australia: Queensland	-19.381	146.449	BOLD IDS
L. reducta MOTU2	LR-05	LOQTB315-07	gvc7053-1L	Australia: Queensland	-19.381	146.449	BOLD IDS
L. reducta MOTU2	LR-06	ANICE507-10	10ANIC-01504	Australia: New South Wales	-35.300	150.240	BOLD IDS
L. reducta MOTU2	LR-06	IMLQ209-07	IM07-0062	Australia: Queensland	-26.444	152.668	BOLD IDS
L. reducta MOTU2	LR-06	IMLQ228-07	IM07-0137	Australia: Queensland	-26.444	152.668	BOLD IDS
L. reducta MOTU2	LR-06	IMLQ448-08	IM07-0118	Australia: Queensland	-26.444	152.668	BOLD IDS
L. binotata MOTU1	LB-01	LOQB572-05	Moth 051.03CL	Australia: Queensland	-17.117	144.533	BOLD IDS
L. binotata MOTU1	LB-01	LOQB615-05	Moth 094.03CL	Australia: Queensland	-17.117	144.533	BOLD IDS
L. binotata MOTU1	LB-04	LOQB619-05	Moth 098.03CL	Australia: Queensland	-17.117	144.533	BOLD IDS
L. binotata MOTU2	LB-02	ANICE510-10	10ANIC-01507	Australia: Northern Territory	-16.080	130.190	BOLD IDS
L. binotata MOTU2	LB-05	ANICE512-10	10ANIC-01509	Australia: Northern Territory	-16.080	130.190	BOLD IDS
L. binotata MOTU3	LB-03	ANICE511-10	10ANIC-01508	Australia: Western Australia	-15.410	128.450	BOLD IDS
*Lymantria antennata**	LA-01	HQ921458	N/A	Australia: Queensland	N/A	N/A	GenBank

*An outgroup. **Published sequences in BOLD IDS and GenBank are freely publicly available for any researcher.

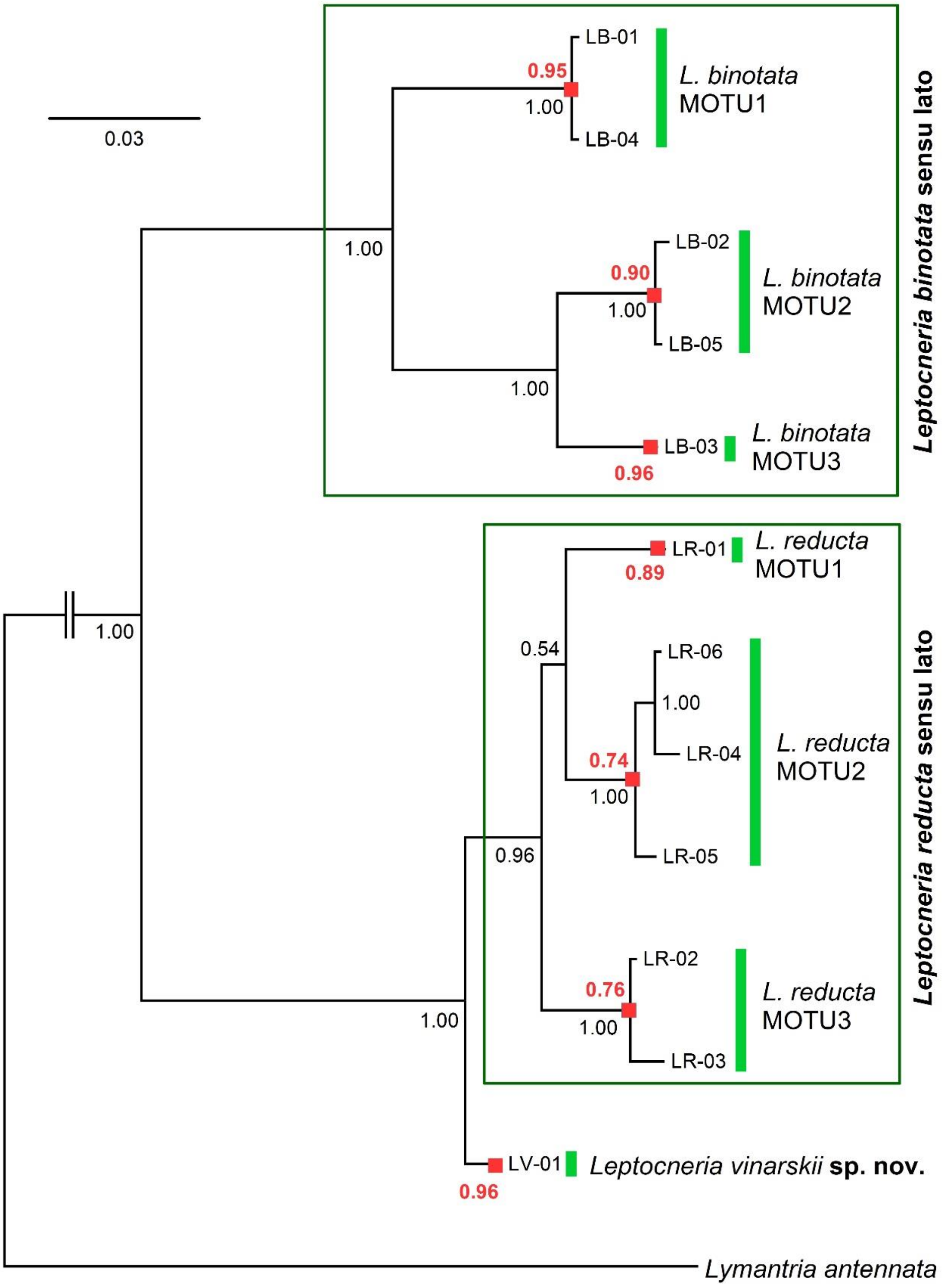

Supplementary Figure 1. Fifty-percent majority-rule consensus phylogenetic tree of *Leptocneria* spp. recovered from Bayesian analysis of the *COI* dataset with 12 in-group haplotypes (see Supplementary Table 1 for details). A haplotype of *Lymantria antennata* was used as an out-group. Black numbers near branches are Bayesian posterior probabilities. Solid red numbers near nodes are probabilities of species-level MOTUs (red squares) based on the highest Bayesian supported solution of the PTP species delimitation model. Scale bar indicates the branch lengths (nucleotide substitutions per site).

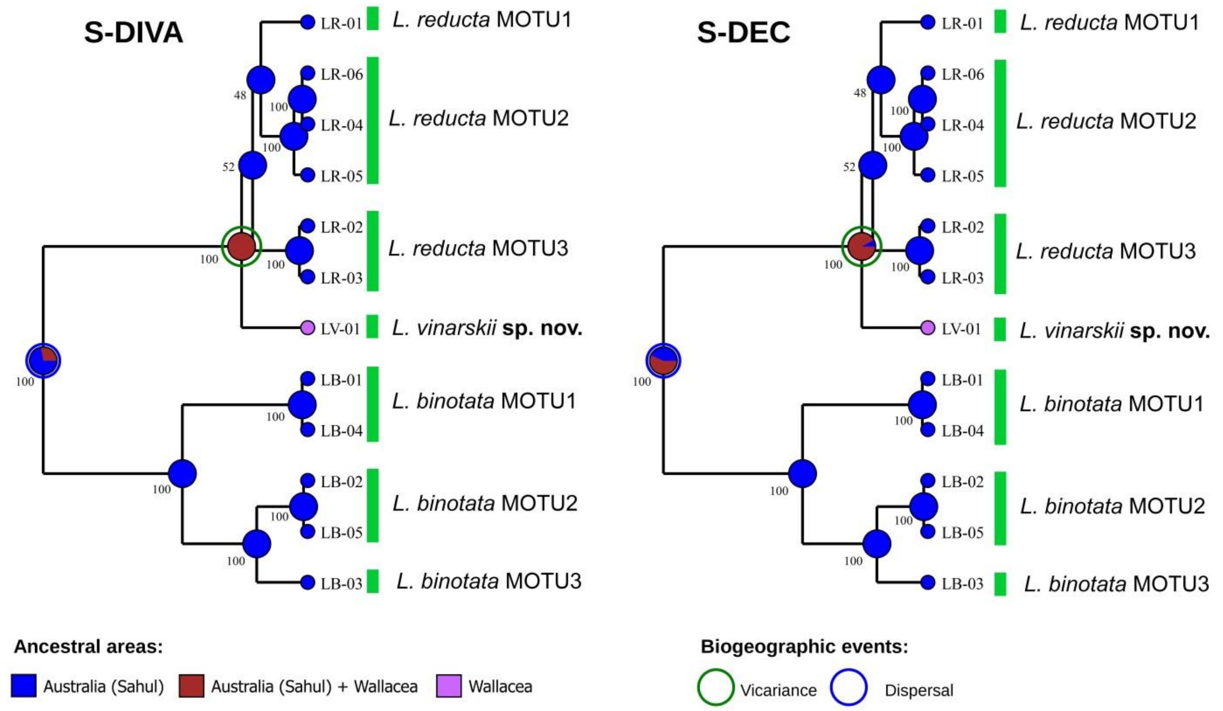

Supplementary Figure 2. Historical biogeography of the genus *Leptocneria* inferred from two different modeling approaches (S-DIVA and S-DEC). The ultrametric chronogram was calculated under a lognormal relaxed clock model and a Yule process speciation implemented in BEAST 2.4.6 and was obtained for the *COI* dataset with 12 in-group haplotypes (see Supplementary Table 1 for details). Pie chaps near nodes indicate the probabilities of certain ancestral areas. Black numbers near nodes are Bayesian posterior probabilities inferred from BEAST.
